# Pancreatic Exocrine Enzymes and Intrapancreatic Protein Synthesis in Acute Oedematous Pancreatitis

**DOI:** 10.1155/1994/25496

**Published:** 1994

**Authors:** Yoshio Kinami, Ichiro Kita, Yasuhiko Kojima, Shigeki Takashima

**Affiliations:** 1 Division of Cancer Research Medical Research Institute Kanazawa Medical University Ishikawa Uchinada Japan; 2 Department of Surgery II Kanazawa Medical University Ishikawa Uchinada Japan; 3 Division of Cancer Research Medical Research Institute Kanazawa Medical University Uchinada-Machi, Kahoku-Gun, Ishikawa 920-02 Japan

## Abstract

Changes in serum and intrapancreatic enzyme content and protein synthesis in pancreas were studied in acute oedematous pancreatitis (AOP). Male Wistar rats (*n* = 111) were divided into 2 groups, controls with a sham operation and those with AOP. Serum amylase levels rose immediately after the procedure causing AOP and then fell gradually, while serum lipase and ribonuclease levels remained higher than control values over 48h. (*p* < 0.05, 0.01). Serum deoxyribonuclease (DNase) II levels were unchanged. Intrapancreatic enzyme levels were scarcely affected by AOP. ^3^H-leucine uptake into pancreatic tissue of rats with AOP was decreased throughout the study (*p* < 0.001), but some protein synthesis continued. Intrapancreatic enzyme contents are maintained despite diffusion into the blood because the pancreas retain its ability to synthesize enzymes.


*HPB Surgery*, 1994, Vol. 8, pp. 43–48
Reprints available directly from the publisher
Photocopying permitted by license only

© 1994 Harwood Academic Publishers GmbH
Printed in Malaysia

### Pancreatic Exocrine Enzymes and Intrapancreatic Protein Synthesis in Acute Oedematous Pancreatitis

YOSHIO KINAMI[1,2], ICHIRO KITA[2], YASUHIKO KOJIMA[2] and SHIGEKI TAKASHIMA[2]

Division of Cancer Research, Medical Research Institute[1], and Department of Surgery II[2], Kanazawa Medical University, Uchinada, Ishikawa, Japan

Changes in serum and intrapancreatic enzyme content and protein synthesis in pancreas were studied in acute oedematous pancreatitis (AOP). Male Wistar rats ($n = 111$) were divided into 2 groups, controls with a sham operation and those with AOP. Serum amylase levels rose immediately after the procedure causing AOP and then fell gradually, while serum lipase and ribonuclease levels remained higher than control values over 48 h. ($p < 0.05, 0.01$). Serum deoxyribonuclease (DNase) II levels were unchanged. Intrapancreatic enzyme levels were scarcely affected by AOP. $^3$H-leucine uptake into pancreatic tissue of rats with AOP was decreased throughout the study ($p < 0.001$), but some protein synthesis continued. Intrapancreatic enzyme contents are maintained despite diffusion into the blood because the pancreas retain its ability to synthesize enzymes.

KEY WORDS: Acute oedematous pancreatitis protein synthesis pancreatic enzymes deoxyribonuclease

### INTRODUCTION

Acute oedematous pancreatitis (AOP) is classified as an attack with mild or moderate clinical features, which may develop into a severe form with parenchymal necrosis or haemorrhage[1]. Several workers have studied the diffusion of exocrine enzymes from the pancreas to the blood[2–4] or changes in protein and enzyme synthesis in exocrine cells[5–7], including some investigations on experimental pancreatitis[8–10].

No elevation of exocrine enzymes in the blood may occur throughout the attack in patients with severe haemorrhagic or necrotizing pancreatitis[11–13], while in those with AOP enzyme levels rise in the absence of pancreatic necrosis[14]. The relationship between serum and intrapancreatic enzyme levels and pancreatic protein synthesis in AOP was investigated in the persent study.

*Address for correspondence*: Dr. Y. Kinami, Division of Cancer Research, Medical Research Institute, Kanazawa Medical University, Uchinada-Machi, Kahoku-Gun, Ishikawa 920-02, Japan.

### MATERIALS AND METHODS

### *Operations and samples*

One hundred and eleven male Wistar rats weighing 200–300 g were given a pellet diet and water ad libitum and were fasted for 24 h before the experiments. Sixty-two rats (the AOP group) underwent laparotomy under ether anaesthesia with ligation of the lower bile duct using Block's method[15]. Forty-two rats (controls) underwent a sham operation. Another 7 normal rats were simply fasted for 24 h.

Twelve rats in each group were used to examine only the 48 h mortality rates. Blood was taken quickly form the abdominal aorta of 7 rats in each group 12, 24, 36 and 48 h after the procedure. After saline lavage of the abdominal cavity the pancreas was removed. Likewise, blood samples and the pancreas were obtained from normal rats. Half the pancreas from 2 rats in each group was used to prepare histological sections stained with hematoxylin eosin.

*Pancreatic enzyme assays*

Five rats from each group (including normal rats) were used for pancreatic enzyme assay. Blood samples were centrifuged and serum obtained. Half the pancreas was homogenized at 0°C in 10 ml saline. The homogenate was centrifuged at 0°C for 20 min at 10,000xg, and the supernatant was obtained. Pancreatic enzyme levels were measured in serum or supernatant as follows. Amylase was determined by an ultraviolet method and expressed in somogyi U per ml serum or mg pancreas protein. Lipase was determined by the Marupi Lipase Kit (Dainippon Pharmaceutical Co.) and was expressed in IU per 1 serum or mg pancreas protein. Ribonuclease (RNase) was determined by Reddi's method[16], using polycytidylic acid (P–L Biochemicals Inc.) as the substrate, and the absorbance was determined at 278 nm; levels were expressed as U per ml serum or mg pancreas protein. Deoxyribonuclease (DNase) II was determined as follows. To 0.5 ml sample was added 0.5 ml DNA solution (4 mg/ml; Sigma Chemical Co.) as substrate plus 2.5 ml 0.2 M pH 5.0 acetic acid buffer containing 20 mM $Mg^{2+}$. This mixture was allowed to react at 35°C for 2 h before. The reaction was stopped by adding 0.5 ml 40% perchloric acid; the reaction mixture was centrifuged at 12,000 xg for 15 min. The supernatant was treated by Burton's method[17], and the absorbance was determined at 600 nm. DNase II level in the samples was expressed as U per 0.5 ml serum or mg pancreas protein. Protein estimations in pancreatic tissue were performed by Lowry's method[18].

*Amino acid uptake*

Seven rats from each group (including normal rats) were used to determine the pancreatic uptake of labelled amino acid. Before sacrifice, $3.7 \times 10^3$ Bq/body wt $^3$H-leucine (DL-[4.5-$H^3$] leucine, specific activity; 0.92–1.85 TBq/mmol) was injected into the tail vein. Half the pancreas was homogenized at 0°C in 10 ml 0.05 M Tris-HCl buffer (pH 7.0). The homogenate was centrifuged at 0°C for 20 min at 10,000 xg, and 4 ml supernatant was centrifuged at 3,000 xg for 10 min after the addition of 1 ml 25% trichloroacetic acid (TCA). TCA-soluble and TCA-insoluble fractions were assessed for radioactivity in a liquid scintillation counter after the addition of a scintillater and its level was expressed as dpm/mg pancreas protein. The percentage of radioactivity in the TCA-insoluble fraction of the homogenate was used to measure the incorporation rate of isotope.

*Statistics*

All data were presented as means ± SD, and Student's *t*-test was used to compare data between groups.

### RESULTS

*Mortality rates and histological findings*

All controls undergoing the sham operation survived throughout the study period. In the AOP group mortality rates were 1/12 at 12 h, 2/12 at 24 h, 3/12 at 36 h and 4/12 at 48 h. No histological changes were observed in control pancreas. At 12 h in the AOP group the pancreas revealed oedema, inflammatory cell infiltration and slight interstitial haemorrhage. By 24 h, these changes had progressed and were accompanied by a slight decrease in zymogen granules in the exocrine cells. At 48 h the pancreas exhibited intracellular vacuoles and cell necrosis.

*Serum enzyme levels (Table 1)*

Controls showed no changes in serum amylase, but in rats with AOP amylese was elevated at 12 h before

**Table 1** Pancreatic enzyme levels[a] in the serum after acute oedematous pancreatitis (AOP)

| | | | *After procedures* | | | |
|---|---|---|---|---|---|---|
| *Enzymes* | *Groups* | *Before* | *12 h* | *24 h* | *36 h* | *48 h* |
| Amylase | Control | 21 ± 5 | 23 ± 7 | 22 ± 11 | 21 ± 10 | 21 ± 9 |
| (somogyi U/ml) | AOP | 21 ± 5 | 38 ± 10[b] | 34 ± 9 | 25 ± 16 | 16 ± 8 |
| Lipase | Control | 26 ± 9 | 40 ± 21 | 33 ± 21 | 46 ± 33 | 33 ± 24 |
| (IU/l) | AOP | 26 ± 9 | 256 ± 45[c] | 267 ± 35[c] | 269 ± 27[c] | 222 ± 67[c] |
| RNase | Control | 50 ± 4 | 53 ± 2 | 54 ± 2 | 53 ± 2 | 52 ± 2 |
| (U/ml) | AOP | 50 ± 4 | 410 ± 155[c] | 390 ± 128[c] | 310 ± 150[c] | 274 ± 168[c] |
| DNase II | Control | 623 ± 174 | 628 ± 123 | 655 ± 175 | 688 ± 108 | 629 ± 129 |
| (U/0.5 ml) | AOP | 623 ± 174 | 582 ± 103 | 736 ± 127 | 694 ± 233 | 583 ± 103 |

[a] All data; mean ± SD, $n = 5$. [b] $p < 0.05$, [c] $p < 0.01$ vs. control group.

**Table 2** Pancreatic enzyme levels[a] in pancreatic tissue after acute oedematous pancreatitis (AOP).

| | | | *After procedures* | | | |
|---|---|---|---|---|---|---|
| *Enzymes* | *Groups* | *Before* | *12 h* | *24 h* | *36 h* | *48 h* |
| Amylase | Control | 19 ± 5 | 18 ± 4 | 18 ± 5 | 18 ± 5 | 18 ± 4 |
| (somogyi U/mg protein) | AOP | 19 ± 5 | 18 ± 4 | 16 ± 4 | 15 ± 4 | 16 ± 4 |
| Lipase | Control | 55 ± 4 | 56 ± 4 | 55 ± 4 | 55 ± 4 | 55 ± 4 |
| (IU/mg protein) | AOP | 55 ± 4 | 54 ± 4 | 52 ± 4 | 52 ± 4 | 49 ± 2[b] |
| RNase | Control | 43 ± 18 | 42 ± 17 | 44 ± 14 | 46 ± 16 | 43 ± 19 |
| (U/mg protein) | AOP | 43 ± 18 | 39 ± 17 | 34 ± 15 | 39 ± 12 | 42 ± 16 |
| DNase II | Control | 23 ± 5 | 22 ± 5 | 23 ± 7 | 22 ± 7 | 22 ± 7 |
| (U/mg protein) | AOP | 23 ± 5 | 23 ± 7 | 24 ± 7 | 20 ± 7 | 20 ± 8 |

[a] All data; mean ± SD, $n = 5$. [b] $p < 0.05$ vs. control group.

returning to control level. Control lipase levels rose slightly after operation, but in rats with AOP lipase was substantially higher throughout the experiment. Likewise, RNase levels remained constant in controls, but were greatly increases throughout in rats with AOP. Serum DNase II levels were unaltered in either group.

### *Intrapancreatic enzyme levels (Table 2)*

Amylase levels were unchanged in controls on rats with AOP. The control procedure caused no enzyme changes. Amylase, RNase and DNase II levels were not altered in AOP, but lipase levels fell by 48 h.

### *Amino acid uptake into pancreatic tissue (Table 3)*

Radioactivity in the TCA-soluble fraction of pancreatic tissue was not significantly altered in controls compared to normal rats, but in the AOP group the 48 h value was lower than controls. Radioactivity in the TCA-insoluble fraction in the control group fell below values of normal rats at 12, 24, and 36 h, but in rats with AOP radioactivity was only about a quarter of control values throughout the experiment. Generally the changes in radioactivity in the homogenate mirrored those seen in the TCA-insoluble fraction. Likewise, incorporation rates were much lower after AOP or all time points.

### DISCUSSION

The interstitial type of acute pancreatitis, the commonest form, can be distinguished from the necrotizing haemorrhagic type on pathological findings and clinical features[19,20]. Clarifying the relationship between changes in serum and intrapancreatic enzymes and protein synthetic capacity in the pancreas should allow rational anti-enzyme therapy. The experimented model of AOP in rats has previously been used[15,21,22], and Block's technique is a simple means of obtaining an uniform model[15]. The mortality rates and histological changes were similar to those reported elsewhere[21,23]. The early rise and subsequent fall in serum amylase has been observed both clinical[2,13,14] and experimentally[9,10,23]. The fall in serum amylase could reflect the action of an inhibitor[24] or its short half life[25].

**Table 3** $^3$H-leucine uptake in pancreatic tissue after acute oedematous pancreatitis (AOP)[a].

| *Fractions and incorporation rates* | | | *After procedures* | | | |
|---|---|---|---|---|---|---|
| | *Groups* | *Before* | *12 h* | *24 h* | *36 h* | *48 h* |
| TCA soluble[b] | Control | 6.4 ± 1.0 | 5.7 ± 0.8 | 5.0 ± 1.1 | 6.5 ± 1.2 | 7.2 ± 1.7 |
| | AOP | 6.4 ± 1.0 | 4.7 ± 1.0 | 5.3 ± 1.8 | 5.0 ± 2.9 | 4.9 ± 1.5[f] |
| TCA insoluble[b] | Control | 28.6 ± 5.0 | 22.3 ± 3.7[d] | 20.5 ± 4.0[d] | 20.4 ± 2.6[e] | 22.2 ± 6.6 |
| | AOP | 28.6 ± 5.0 | 4.3 ± 5[g] | 5.3 ± 1.8[g] | 4.9 ± 1.8[g] | 5.2 ± 1.0[g] |
| Homogenate[b] | Control | 35.0 ± 4.5 | 28.0 ± 4.4 | 25.5 ± 4.1 | 26.9 ± 2.8 | 29.4 ± 7.1 |
| | AOP | 35.0 ± 4.5 | 9.0 ± 1.9[g] | 10.6 ± 2.4[g] | 9.9 ± 4.6[g] | 10.1 ± 2.2[g] |
| Incorporation | Control | 82 ± 5 | 80 ± 1 | 80 ± 1 | 76 ± 4 | 76 ± 7 |
| rates[c] | AOP | 82 ± 5 | 48 ± 9[g] | 50 ± 12[g] | 50 ± 10[g] | 52 ± 8[g] |

[a] All data; mean ± SD, $n = 7$. [b] Radioactivities; $\times 10^2$ dpm/mg protein. [c] Rates; %. [d] $p < 0.05$, [e] $p < 0.01$ vs. before procedure. [f] $p < 0.05$, [g] $p < 0.001$ vs. control group.

The more prolonged duration of raised serum lipase has already been reported[9,10,23].

RNase has been detected in the serum, pancreatic tissue and pancreatic juice of patients with pancreatitis or pancreatic cancer by many investigators[26,27]. Warshaw *et al.*[28] reported an obvious rise in serum RNase levels of patients with pancreatic necrosis or abscess. In this experiment, serum RNase rose sharply in the early stage of pancreatitis and then fell gradually, though (unlike amylase) levels remained elevated for 48 h. There are 4 types of DNase[29]. Acid DNase relates to the metabolism of intracellular nucleic acid, and neutral DNase was secreted into pancreatic juice as a digestive enzyme[30]. This experiment revealed no increase in serum DNase II levels (the acid type). Within the pancreas itself there was very little change in levels of any enzyme.

Most of the proteins synthesized in the pancreas are exocrine enzymes. Many studies of pancreatic protein synthesis have used incorporation of labelled amino acids[5-8,31]. Since autoradiographic studies in rodents have shown that most grains are seen on zymogen granules after 1 h, $^{3}$H-leucine uptake into the pancreas was examined at 1 h. Whereas radioactivity in the TCA soluble fraction fell slightly in controls and then recovered, values in AOP group persisted at a slightly lower level. The control operation reduced radioactivity in the TCA insoluble fraction but AOP produced a profound and persistent fall. The decrease in amino acid incorporation shows that there was reduced enzyme synthesis, though some activity continued during AOP.

In summary, serum alterations in exocrine enzyme levels did not clearly reflect changes in pancreatic enzyme content, nor did it always indicate the pathological changes in the pancreas[11-14]. Since enzyme synthesis is maintained within the pancreas, the results suggest the necessity for continuing anti-enzyme therapy in patients with AOP.

### REFERENCES

1. Singer, M. V., Gyr, K. and Sarles, H. (1985) Revised classification of pancreatitis, report of the second international symposium on the classification of pancreatitis in marseille, france, March 28–30, 1984. *Gastroenterology*, **89**, 683–690.
2. Elman, R., Ameson, N. and Graham, E. A. (1929) Value of blood amylase estimation in the diagnosis of pancreatic disease, a clinical study. *Arch. Surg.*, **19**, 943–967.
3. Creutzfeldt, W. and Schmidt, H. (1970) Aetiology and pathogenesis of pancreatitis. *Scand. J. Gastroenterol.*, **5** (suppl. 6), 47–62.
4. Ishihara, Y. (1988) Laboratory studies of the pancreatic disease—significance of serum pancreatic enzymes determination. *J. Clin. Exper. Med.*, **144**, 395–398.
5. Warshawsky, H., Leblond, C. P. and Droz, B. (1963) Synthesis and migration of proteins in the cells of the exocrine pancreas as revealed by specific activity determination from radioautographs. *J. Cell Biol.*, **16**, 1–23.
6. Caro, L. G. and Palade, G. E. (1964) Protein synthesis, storage, and discharge in the pancreatic exocrine cell, an autoradiographic study. *J. Cell Biol.*, **20**, 473–495.
7. Jamieson, J. D. and Palade, G. E. (1968) Intracellular transport of secretory proteins in the pancreatic exocrine cell. III. Dissociation of intracellular transport from protein synthesis. *J. Cell Biol.*, **39**, 580–588.
8. Ekholm, R., Edlund, Y. and Zelander, T. (1962) The ultrastructure of the rat exocrine pancreas after brief ethionine exposure. *J. Ultrastruct. Res.*, **7**, 102–120.
9. Takita, Y. (1980) Histological and functional changes of the pancreas after experimental acute pancreatitis. *J. Juzen Med. Soc.*, **89**, 187–202.
10. Sugawara, S. (1990) The influence of pancreatic neurotomy upon acute pancreatitis in rats. *J. Juzen Med. Soc.*, **99**, 588–603.
11. Kaplan, M., Cotlar, A. M. and Stagg, S. J. (1964) Acute pancreatitis, six year survey with evaluation of steroid therapy. *Am. J. Surg.*, **108**, 24–30.
12. Jacobs, M. L., Daggett, W. M., Civetta, J. M., Andrevasu, M., Lawson, D. W., Warshaw, A. L., Nardi, G. L. and Bartlett, M. K. (1977) Acute pancreatitis: analysis of factors influencing survival. *Ann. Surg.*, **185**, 43–51.
13. Ogawa, M., Matsuda, K., Matsuda, Y., Miyauchi, K., Nishijima, J., Horikawa, Y., Kurihara, M. and Mori, T. (1985) Evaluation of immunological assays of serum pancreatic enzymes and pancreatic secretory trypsin inhibitor for diagnosis and management of acute pancreatitis. In Pancreatitis, Its Pathophysiology and Clinical Aspects, edited by T. Sato and H. Yamauchi, pp. 107–115, Tokyo: University of Tokyo Press.
14. Kinami, Y., Miyazaki, I., Konishi, K. and Noguchi, M. (1980) Clinical features and therapy of acute pancreatitis. *Geka*, **42**, 551–557.
15. Block, M. A., Wakim, K. G. and Baggenstoss, A. H. (1954) Experimental studies concerning factors in the pathogenesis of acute pancreatitis. *Surg. Gynecol. Obstet.*, **99**, 83–90.
16. Reddi, K. K. (1975) Nature and possible origin of human serum ribonuclease. *Biochem. Biophys. Res. Commun.* **67**, 110–118.
17. Burton, K. (1956) A study of the conditions and mechanism of the diphenylamine reaction for the colorimetric estimation of deoxyribonucleic acid. *J. Biochem.*, **62**, 315–323.
18. Lowry, O. H., Roserbrough, N. J., Farr, A. L. and Randall, R. J. (1951) Protein measurement with folin phenol reagent. *J. Biol. Biochem.*, **193**, 265–275.
19. Baggenstoss, A. H. (1973) Pathology of pancreatitis. In Pancreatitis, edited by E. E. Gambill, pp. 176–219, Saint Louis: C. V. Mosby Co.
20. Tuzhilin, S. A. (1974) The clinical features of pancreatic inflammation. *Am. J. Gastroenterol.* **61**, 97–112.
21. Shinmura, K. (1979) Experimental studies on inhibition of pancreatic protein synthesis—treatment of acute pancreatitis and prevention of post-operative pancreatic complications caused by pancreatic enzymes—. *J. Juzen Med. Soc.*, **88**, 882–895.
22. Kinami, Y. (1983) Experimental acute pancreatitis. *Biliary Tract and Pancreas*, **4**, 1325–1332.
23. Kinami, Y. and Kita, I. (1989) Relationship between pancreatic enzymes and pathological changes in the pancreas in acute pancreatitis, the significance of determination of serum deoxyribonuclease. *Inter. J. Pancreatol.*, **4**, 371–381.
24. Ogawa, M., Takatsuka, Y., Kitahara, T., Matsuura, K. and Kosakai, G. (1981) Radioimmunoassay of human pancreatic amylase. *Meth. Enzymol.*, **74**, 290–298.
25. Duane, W. C., Freriche, R. and Levitt, M. D. (1972) Simultaneous study of the metabolic turnover and renal excretion of salivary amylase-$^{125}$I and pancreatic amylase-$^{131}$I in the baboon. *J. Clin. Invest.*, **51**, 1504–1513.

26. Zendzian, E. N. and Barnard, E. A. (1967) Distributions of pancreatic ribonuclease, chymotrypsin, and trypsin in vertebrates. *Arch. Biochem. Biophys.*, **122**, 699–713.
27. Reddi, K. K. and Holland, J. F. (1976) Elevated serum ribonuclease in patients with pancreatic cancer. *Natl. Acad. Sci. USA*, **7**, 2308–2310.
28. Warshaw, A. L. and Lee, K. H. (1979) Serum ribonuclease elevations and pancreatic necrosis in acute pancreatitis. *Surgery*, **86**, 227–234.
29. Lindall, T., Gally, J. A. and Edleman, G. M. (1969) Properties of deoxyribonuclease III from mammalian tissue. *J. Biol. Chem.*, **18**, 5014–5019.
30. Allfrey, V. and Mirsky, E. (1952) Some aspect of the deoxyribonuclease activities of animal tissue. *J. Gen. Physiol.*, **36**, 227–241.
31. Palade, G. E. (1975) Intracellular aspect of the process of protein synthesis. *Science*, **189**, 347–358.
32. Kinami, Y., Kawamura, M., Sugii, M. and Shinmura, K. (1975) Effects of anticancer drugs on protein synthesis of the exocrine pancreas. *Japanese J. Gastroenterol.*, **72**, 811–821.

### INVITED COMMENTARY

Although progress towards an effective therapy for acute pancreatitis has been limited by several factors, notably the absence of an ideal experimental model[1,2], considerable advances have been made in the detection and treatment of the complications of the disease. Unfortunately, these advances have not been paralleled by much improvement in understanding the basic pathophysiology of the disease.

In this study, Dr Kinami and his colleagues try to clarify the relationship between serum enzymes, intrapancreatic enzymes and pancreatic protein synthesis, a relationship that has been extensively studied over the past decade. Their results generally confirm previous publications on the subject, namely that serum enzyme levels do not accurately reflect the pathological changes in the gland and that enzyme synthesis persists, at least during the early phases of the disease process[3].

The authors noted a slight decrease in acinar zymogen granule concentration at 24 h. This finding differs from most experimental models of acute pancreatitis, in which intracellular zymogen granule concentration is increased at an early stage[8] due to impaired acinar cell secretion[4,5,6,7]. Moreover, recent data suggest that digestive zymogen activation occurs within the acinar cell itself[9,10]. Thus, drugs directed at inhibiting acinar cell secretion, as a means of controlling pancreatitis, may actually worsen the disease by increasing intracellular zymogen concentration.

Most researchers now believe that intracellular enzyme activation proposed by Rao *et al.*[11] is a more crucial factor in the pathogenesis of acute pancreatitis than enzyme synthesis per se. Therefore, although anti-enzymatic therapy may be indicated in the treatment of acute pancreatitis, it should probably not be directed towards stopping enzyme synthesis, as suggested by the authors in their concluding remarks, but rather towards stopping intracellular enzyme activation. Indeed, clinical trials on the systemic administration of the antiprotease aprotinin (Trasylol) have proved ineffective[12].

Newer studies on the pathogenesis of acute pancreatitis suggest that autoactivation of trypsinogen is the responsible factor for initiating intracellular zymogen activation[8]. This process requires a low pH of around 5[9] and probably takes place in newly formed intracellular vacuoles[7,13,14], which appear as a result of an abnormal subcellular distribution of digestive zymogens and lysosomal hydrolases during the early phases of the disease[3]. The vacuoles noted on histological sections of the pancreas at 48 h in the present study (although reported to occur in experimental models involving diet and secretagogue-induced pancreatitis) were not usually noted in similar models of pancreatic duct obstruction[3]. Their appearance characterizes an early stage of the disease in many animal models and possibly in human disease[15].

In conclusion we believe that to reach the authors' final suggestion about the "necessity of anti-enzymatic therapy to be carried out continuously on patients with acute oedematous pancreatitis", a number of important question remains to be answered: What enzyme, or combination of enzymes, should we aim at counteracting and what are the objective means of selecting our potential patients, i.e., the minority who may progress from acute oedematous pancreatitis to the necrotizing or haemorrhagic forms of the disease?

It is hoped that cellular models of acute pancreatitis will provide some answers to similar questions in the near future.

G. A. Nawfal
RCN Williamson
Directorate of Surgery
Hammersmith Hospital
London W12 ONN
U.K.

### REFERENCES

1. Steinberg, W. A. and Schlesselman, S. E. (1987) Treatment of pancreatitis: Comparison of animal and human studies. *Gastroenterology*, **93**, 1420–7.
2. Bilchik, A. J., Leach, SD., Zucker, K. A. and Modlin, I. M. (1990) Experimental models of acute pancreatitis. *J. Surg. Res.* **48**, 639–47.
3. Saluja, A., Saluja, M., Villa, A., Leli, U., Rutledge, P., Meldolesi, J. and Steer, M. (1989) Pancreatic duct obstruction in rabbits causes digestive zymogen and lysosomal enzyme colocalization. *J. Clin. Invest.* **84**, 1260–6.
4. Niederau, C., Niederau, M. and Luthen, R. *et al.* (1990) Pancreatic exocrine secretion in acute experimental pancreatitis. *Gastroenterology*, **99**, 1120–7.
5. Evander, A., Hederstrom, E., Hultberh, B. and Ihase, I. (1982) Exocrine pancreatic secretion in acute experimental pancreatitis. *Digestion*, **24**, 159–67.
6. Kimura, T., Zuidema, G. D. and Cameron, J. L. (1980) Experimental pancreatitis: Influence of the secretory state of the pancreas. *Surgery*, **88**, 661–6.
7. Steer, M. L. and Meldolesi, J. (1987) The cell biology of experimental pancreatitis. *N. Engl. J. Med.* **316**, 144–50.
8. Leach, S. D., Gorelick, F. S. and Modlin, I. M. (1992) New perspectives on acute pancreatitis. *Scand. J. Gastroenterol.* **27**, (suppl 192), 29–38.
9. Leach, S. D., Modlin, I. M., Scheele, G. A. and Gorelick, F. S. (1991) Intracellular activation of digestive zymogens in rat pancreatic acini: Stimulation by high doses of cholecystokinin. *J. Clin. Invest.* **87**, 362–6.
10. Bialek, R., Willemer, S., Arnold, R. and Adler, G. (1991) Evidence of intracellular activation of serine proteases in acute cerulein-induced pancreatitis in rats. *Scand. J. Gastroenterol.* **26**, 190–6.
11. Rao, K. N., Zuretti, M. F., Baccino, F. M. and Lombardi, B. (1980) Acute hemorrhagic pancreatic necrosis in mice: The activity of lysosomal enzymes in the pancreas and the liver. *Am. J. Pathol.* **98**, 45–59.
12. Imrie, C. W., Benjamin, I. S. and Ferguson, J. C. (1978) A single center double blind trial of trasylol therapy in primary acute pancreatitis. *Br. J. Surg.* **65**, 337–41.
13. Niederau, C. and Grendell, J. H. (1988) Intracellular vacuoles in experimental acute pancreatitis in rats and mice are an acidified compartment. *J. Clin. Invest.* **81**, 229–36.
14. Leach, S. D., Karapetian, O., Den Ouden, L. B., Marino, C. M., Modlin, I. M. and Gorelick, F. S. (1989) Hyperstimulation of isolated pancreatic acini results in crinophagic vacuoles with an acidic internal pH. **4**, Pancreas, 627.
15. Kloppel, G., Dreyer, T., Willemer, S., Kern, H. F. and Adler, G. (1986) Human acute pancreatitis: Its pathogenesis in the light of immunocytochemical and ultrastructural findings in acinar cells. *Virchows. Arch.* (*A*) **409**, 791–803.

